# The Validity of Functional Near-Infrared Spectroscopy Recordings of Visuospatial Working Memory Processes in Humans

**DOI:** 10.3390/brainsci8040062

**Published:** 2018-04-05

**Authors:** Joëlle S. Witmer, Eva A. Aeschlimann, Andreas J. Metz, Stefan J. Troche, Thomas H. Rammsayer

**Affiliations:** 1 Institute of Psychology, University of Bern, 3012 Bern, Switzerland; joelle.witmer@psy.unibe.ch (J.S.W.); eva.aeschlimann@psy.unibe.ch (E.A.A.); andreas.j.metz@gmail.com (A.J.M.); 2 Department of Psychology and Psychotherapy, University of Witten/Herdecke, 58455 Witten, Germany; stefan.troche@uni-wh.de (S.J.T.)

**Keywords:** fNIRS, visuospatial working memory capacity, validity, sensitivity, specificity, subjective task demand, mental ability, duration discrimination

## Abstract

Functional near infrared spectroscopy (fNIRS) is increasingly used for investigating cognitive processes. To provide converging evidence for the validity of fNIRS recordings in cognitive neuroscience, we investigated functional activation in the frontal cortex in 43 participants during the processing of a visuospatial working memory (WM) task and a sensory duration discrimination (DD) task functionally unrelated to WM. To distinguish WM-related processes from a general effect of increased task demand, we applied an adaptive approach, which ensured that subjective task demand was virtually identical for all individuals and across both tasks. Our specified region of interest covered Brodmann Area 8 of the left hemisphere, known for its important role in the execution of WM processes. Functional activation, as indicated by an increase of oxygenated and a decrease of deoxygenated hemoglobin, was shown for the WM task, but not in the DD task. The overall pattern of results indicated that hemodynamic responses recorded by fNIRS are sensitive to specific visuospatial WM capacity-related processes and do not reflect a general effect of increased task demand. In addition, the finding that no such functional activation could be shown for participants with far above-average mental ability suggested different cognitive processes adopted by this latter group.

## 1. Introduction

In the past 30 years, research on cognitive functioning has been complemented by neuroimaging methods that measure the magnitude of functional activation and the brain areas involved in specific aspects of information processing [1]. The techniques used most frequently in this research area are positron emission tomography (PET) and functional magnetic resonance imaging (fMRI). More recently, functional activation has also been measured with functional near-infrared spectroscopy (fNIRS) [2,3]. The advantage of fNIRS over PET and fMRI is its compact measurement system that is relatively robust to motion artifacts, which enable its use outside of a scanner in a variety of experimental settings. Functional activation requires oxygen, which leads to increased cerebral blood flow due to neurovascular coupling and changes in the local concentrations of two types of hemoglobin (Hb): oxygenated (O_2_Hb) and deoxygenated (HHb) [4]. Near-infrared light of different wavelengths is radiated into the brain tissue where it is absorbed and scattered, and the unabsorbed light is measured. This allows for continuous and noninvasive monitoring and quantification of the concentration changes of O_2_Hb and HHb in the cortex. Several studies comparing fNIRS and fMRI data have reported strong correlations between their signals [5,6,7]. Furthermore, fNIRS is sensitive to cognitive state (e.g., resting state vs. task performance) and cognitive task load [8,9]. These results provide converging evidence that fNIRS is a reliable method that can be used to measure hemodynamic responses in the cortex under specific conditions.

Many neuroimaging studies of information processing have investigated functional activation during the processing of working memory (WM) tasks [6,10,11,12]. WM refers to the capacity-limited system responsible for the simultaneous manipulation and storage of information [13,14,15]. WM capacity is usually assessed with complex span tasks in which the participants are required to remember and mentally process several stimuli over a brief period [16]. However, the use of complex span tasks results in impure measures of WM [17], which indicates that the processing of these tasks requires multiple processes (e.g., perception, attention, storage and motor response). Neuroimaging studies illustrate this with different processes resulting in functional activation in different brain areas (e.g., [18]). A comparison of storage-only and storage-plus-processing tasks conducted by Smith and Jonides [19] revealed functional activation in areas related to the content of a given task and additional functional activation in the prefrontal cortex in storage-plus-processing tasks. The results of a large meta-analysis of WM tasks in fMRI experiments conducted by Rottschy and colleagues [11] had similar conclusions. They suggested that a bilateral frontoparietal core network sustains the basal processes required for most WM-related cognitive functions. Differences in functional activation become apparent according to task-induced cognitive load, task type (e.g., memory of location vs. stimulus identity in visual WM tasks) and type of required recall (e.g., verification, matching or reproduction). For example, tasks requiring memory of location (compared to stimulus identity) and tasks requiring reproduction (compared to verification and matching) result in more functional activation in the posterior superior frontal gyrus [11].

The goal of measuring functional activation resulting exclusively from WM-specific processes has two challenges. The first challenge is the differentiation of functional activation between specific WM-related processes and general WM-unspecific processes, such as stimulus encoding or response selection. To address this first problem, an experimental condition that is able to disentangle WM-specific and WM-unspecific processes is needed [20]. One possible solution for this issue is the so-called subtraction approach [21], which involves an active control condition in which most perceptual and response-related processes are the same as those in the experimental condition, while the WM-specific process of interest is only required for the experimental task. Thus, the differences in functional activation between the experimental and active control conditions can then be attributed to the WM-specific processes of interest.

If, based on the subtraction approach, functional activation can be assigned to the WM-specific processes in the experimental condition, a second challenge emerges. More precisely, it remains unclear whether the observed differences in functional activation between the active control condition and experimental condition reflect the specific WM-related processes or a more general increase in task demand. Therefore, ensuring that the observed changes in functional activation are indeed caused by the specific WM-related task demands rather than by a general increase in task demand, which can be elicited by any task, is important.

To distinguish functional activation induced by a specific WM-related process from that induced by general task-independent effects of increasing task demand, an additional comparison task that is functionally unrelated to WM and that has a task demand comparable to that of the WM task is required. In the present study, we chose to use duration discrimination (DD) as the comparison task. With this task, the participant is required to watch to two successively presented light flashes and to decide which light flash was longer. The discrimination of very brief intervals in the range of milliseconds has been proposed to depend on sensory-perceptual rather than higher-order cognitive processes [22,23]. This suggestion was confirmed by experimental [24,25] and neuropharmacological studies (for a concise review, see [26]), neurocomputational approaches [27] and neuroimaging studies [28]. In line with these results is a meta-analysis that showed that processes involving timing in the range of milliseconds mainly recruit subcortical networks in the basal ganglia and cerebellum [29].

To enable comparisons of the functional activation produced by two different tasks, ensuring that the subjective level of task difficulty for a given individual, and thus the level of task demand elicited by the two tasks, is about the same for both tasks is essential. This can be achieved by using an adaptive procedure to adjust the individual variability of performance across different tasks to a set criterion level [30,31]. Even more importantly, adopting a subjective level of task difficulty is a crucial prerequisite for comparing task-specific functional activation resulting from the same task in multiple individuals. This is particularly important as the interindividual variability of performance within a sample can be large. The lack of a control for a subjective level of task difficulty inevitably implies that some individuals will experience increased task demands, while others will experience decreased task demands. These differences in task demand can produce large differences in functional activation, even within a given experimental condition [32]. From this perspective, the adaptive approach ensures that all participants process the task with subjectively similar levels of task difficulty. Thus, the task demands required to solve the task should be comparable among individuals.

Various neuroimaging studies have reported negative correlations between brain glucose metabolism and mental ability. That is, individuals with higher ability (HA) show lower brain glucose metabolism rates than individuals with lower ability (LA) do when they perform the same cognitive tasks [33] (for a concise review, see [34]). Given the close relationship between mental ability and WM [16,35], it cannot ruled out that also O_2_Hb and HHb concentration changes, as assessed by fNIRS, could be effectively modulated by individual differences in mental ability. For this reason, participants’ individual level of mental ability was controlled for in the present study.

Thus, the major aim of the present study was to investigate whether fNIRS was suitable for measuring WM-specific differences in the concentration changes of Hb oxygenation irrespective of the concurrently ongoing processes that are less specific for WM capacity. More precisely, we probed whether the changes in functional activation observed during the performance of a WM task can be considered specific for WM-related processes rather than subsidiary processes, such as perceptual encoding or motor processes. For this purpose, we compared activation elicited by an experimental WM condition and active control condition. Furthermore, we included DD as an additional task, as well as a corresponding active control condition, which was functionally unrelated to WM capacity. To ensure that the subjective levels of task difficulty were the same for all participants and both tasks, an adaptive procedure was applied for the WM and the DD task.

If the changes in functional activation observed with the WM task were mostly task-unspecific and therefore only reflected the general task demands that were induced by task difficulty, the same pattern of functional activation should be observed during the performances of both the WM and DD tasks. However, if functional activation is observed in a specific cortical area during the performance of the WM task, but not during the performance of the DD task, the functional activation might be directly related to WM capacity. Finally, by comparing two groups of individuals with different levels of mental ability, we assessed how much the functional activation was effectively modulated by individual differences in mental ability.

## 2. Materials and Methods

### 2.1. Participants

In the first session, the participants were screened for mental ability with the German adaptation of Cattell’s Culture Fair Test 20-R [36]. To ascertain the groups with the extremes of mental ability, we calculated that a difference of 16 IQ points was required between the HA and LA groups to avoid any overlap of the 95% confidence intervals. Therefore, we selected study participants with IQs lower than or equal to 112 (LA group: IQ range, 90–112) and IQs higher than or equal to 130 (HA group: IQ range, 130–145). The LA group consisted of 21 participants (11 women; age range, 20–24 years; mean ± standard deviation of age, 21.9 ± 1.5 years), and the HA group consisted of 22 participants (11 women; age range, 18–24 years; mean ± standard deviation of age: 20.8 ± 2.1 years). All participants were right-handed with normal hearing and normal or corrected-to-normal vision. According to self-reports, all were nonsmokers without a history of psychiatric or neurological illness, serious head injury or psychotropic drug use. For participation in the study, they received 100 Swiss Francs. The participants provided written informed consent after they were given detailed explanations of the study protocol and NIRS recording procedures. The study was approved by the ethics committee of the Faculty of Human Sciences of the University of Bern (Bern, Switzerland) (date of approval: 29 July 2014; project identification code: No. 2014-6-880651).

### 2.2. Tasks

#### 2.2.1. Visuospatial WM Task

A combined version of the Matrix Task [37] and Patterns-Memory Task [38] was used to quantify individual visuospatial WM spans. The task was fully computer-controlled and programmed with E-Prime experimental software (version 2.0, Psychology Software Tools, Inc., Sharpsburg, PA, USA). Each trial started with the presentation of a fixation cross for 500 ms and then a black 4 × 4 grid (13.2 × 13.2 cm) with 16 white squares (empty grid) in the center of a touchscreen monitor. In the first condition, two of the 16 squares were successively blackened in a pseudorandom order for 1000 ms each. The participants were required to memorize the location and order of appearance of the black squares. A question mark was then displayed for 1000 ms, and then, an empty grid that was used as an input template for the participant’s response was displayed. The participants were instructed to touch the two squares on the empty grid that were previously black on the touchscreen monitor in reverse order with their right index finger. The task began with a block of four trials with a span of two squares to be memorized. The second block consisted of four trials with a span of three squares to be memorized; the third block consisted of four trials with a span of four squares to be memorized, and so on. The span was increased after each block by one additional square if the participant was able to correctly solve at least three trials within a block. The number of squares in the last edited block in which this criterion was fulfilled corresponded to the individual’s WM span, which was the dependent variable in this task. 

#### 2.2.2. DD Task

To quantify temporal sensitivity in the range of milliseconds, a visual DD task was used. The task was fully computer-controlled and programmed with E-Prime experimental software (Version 2.0, Psychology Software Tools, Inc., Sharpsburg, PA, USA). The stimuli were a 100-ms light flash (the standard interval) and a light flash with variable duration (the comparison interval) that were presented as a computer-controlled red light-emitting diode (diameter, 0.38°; viewing distance, 60 cm; luminance, 68 cd/m^2^) that was positioned at eye level.

The task consisted of 32 trials, with each trial consisting of a standard interval and comparison interval with an interstimulus interval of 900 ms. The duration of the comparison interval varied according to the weighted up-down method [39], which is an adaptive psychophysical procedure. “Adaptive” means that the difference in the stimulus durations between the constant standard stimulus and variable comparison stimulus varied from trial to trial depending on the participant’s previous response. Correct responses resulted in a decrease in the difference between the standard and comparison stimuli, while incorrect responses made the task easier by increasing this difference.

The first comparison interval was 135 ms. During the first six trials, the differences between the standard and comparison intervals were decreased by 5 ms after a correct response and increased by 15 ms after an incorrect response. For the subsequent trials, the step sizes were 3 ms and 9 ms, respectively. After each trial, the participants had to decide whether the first or second interval was longer by pressing one of two designated response keys. Visual feedback was given immediately after the participant’s response was presented for 1500 ms. The next trial started 800 ms after the feedback.

As a psychophysical indicator of performance, the 75%-difference threshold, which was defined as the difference between the standard and comparison stimuli at which a given participant produced 75% correct responses, was calculated. With this procedure, a higher temporal sensitivity was indicated by smaller threshold values. A detailed description of the psychophysical procedure has previously been reported by Rammsayer [40].

#### 2.2.3. fNIRS Tasks

The participants were instructed to sit quietly with their eyes open during the resting phase. All instructions were given orally via headphones (CX 5.00G, Sennheiser electronic GmbH & Co. KG, Wedemark, Germany). Before each task, verbal instructions were given, and then, several practice trials followed. The fNIRS measurements started during a resting phase of 45 s, which was followed by either the DD or WM task. The order of the tasks was counterbalanced across participants. Both tasks consisted of four blocks of the active control task and four blocks of the experimental task. The processing of the experimental tasks required WM-specific (for the visuospatial WM task) and DD-specific (for the DD task) processes, as well as task-unspecific processes, such as attention, perception and encoding of the stimuli, response selection and execution of the motor response. In contrast, the processing of the control tasks consisted of the same processes except for those of the WM-specific and DD-specific processes. With the subtraction approach, this procedure allowed for the identification of WM-/DD-specific changes in Hb oxygenation concentration [20,21,41].

**Figure 1:**
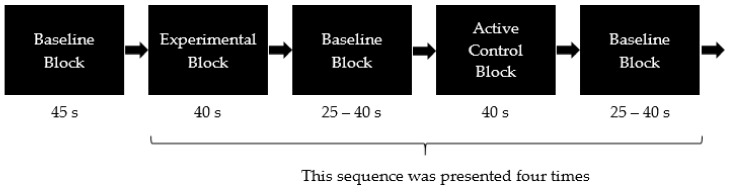
Illustration of the block design, which contained eight baseline blocks (resting phases), four experimental blocks and four active control blocks. After the baseline block, participants started either with an experimental or an active control block (the order was counterbalanced across participants).

The control and experimental blocks were presented in alternating order, and they lasted for 40 s each. All blocks were separated by a resting phase. To reduce the influence of Mayer waves, which are spontaneous oscillations in blood flow that confound fNIRS signals [42] and prevent entrainment of the blood flow changes due to rhythmic changes, the duration of the resting phases were randomly varied from 25–40 s. Within each task, the order of the active control and experimental blocks was also counterbalanced across the participants (see Figure 1).

fNIRS visuospatial WM task: The stimuli and procedures were identical to those in the ordinary WM task, except that no adaptive procedure was applied. Thus, in the experimental condition, each participant performed the task with his/her individual WM span that was determined in the previous experimental session. In the active control condition, the same number of squares was blackened as in the experimental condition, but in a systematic order (from top to bottom or left to right). Thus, the task demands on WM were substantially reduced. The dependent variable, the percentage of correctly solved trials, was calculated.

fNIRS DD task: The stimuli and procedures were identical to those in the ordinary DD task, except that no adaptive procedure was applied and no visual feedback was given. In the experimental condition, the duration of the standard interval was 100 ms, while the comparison interval was 100 ms plus the individual threshold value that was determined in the previous experimental session (e.g., if someone had a discrimination threshold of 28 ms, the duration of the comparison interval would be 128 ms). For the active control blocks, the durations of the standard and comparison intervals were held constant at 100 ms and 360 ms, respectively. This difference in duration between the standard and comparison intervals was well beyond the participants’ DD thresholds and therefore easy to distinguish for all participants. The dependent variable, the percentage of correct responses, was calculated.

### 2.3. fNIRS Recordings

**Figure 2:**
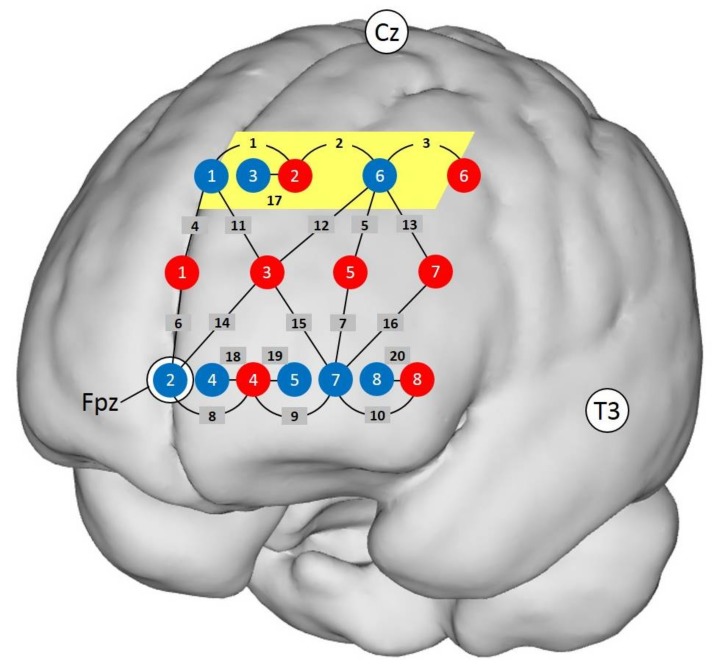
Location of the 20 channels projected on a standard brain. There were eight emitters (red dots) and eight detectors (blue dots). Black numbers indicate channels. The region of interest (ROI) is located in the area of Channels 1, 2 and 3 (highlighted in yellow). Channels 1–10 have a source-detector distance of ~30 mm; Channels 11–16, ~42 mm; Channels 17–20, ~15 mm. Detector 2 was placed at Fpz; Source 1 and Detector 1 on an imaginary line towards Cz; and the bottom row of fibers on an imaginary line towards T3.

The fNIRS data were acquired with a FOIRE-3000 multichannel system (Shimadzu Corporation, Kyoto, Japan) at a sampling rate of 7.69 Hz. The FOIRE-3000 system included eight light-emitter and eight light-detector probes. First, the Nz, Fpz, Cz, Oz, A1, A2 and T3 landmarks were measured and marked on the scalp according to the 10–20 positioning system [43]. The 16 probes were subsequently mounted on the left forehead with a purpose-built probe holder (see Figure S1). As shown in Figure 2, Detector 2 was placed at Fpz, and Source 1 and Detector 1 were located on an imaginary line that extended in the direction of Cz, while the bottom row of the probes (with Sources 4 and 8 and Detectors 2, 4, 5, 7 and 8) were located on an imaginary line that extended in the direction of T3. For each participant, the three-dimensional spatial positions of the marked points on the scalp and each probe in the probe holder were measured using a FASTRAK^®^ 3D magnetic field digitizer (Polhemus, Colchester, VT, USA) and 3D position measurement system software (Version 4.1.0.0, Shimadzu Corporation, Kyoto, Japan). This procedure facilitates the determination of the precise channel positions despite variations in head size and shape [44]. We modified our standard probe holder to allow for the measurement of short channels. With these, the influence of the superficial skin and scalp tissue can be reduced, and the sensitivity for the cortex therefore increases [41]. We arranged the probe holder so that it included four short channels (source-detector distance, ~15 mm), 16 longer channels (source-detector distance, ~30 mm) and six long channels (source-detector distance, ~42 mm). Short channel regression was applied to diminish superficial influences, such as hemodynamic fluctuations in the scalp [45] in both O_2_Hb and HHb [46]. Each long channel was regressed with the closest short channel (see Figure 2). The short channel regression was only applied if the correlation between the time series of the two signals was sufficient such that *R*^2^ > 0.1, which is a criterion adapted from Gagnon et al. [47]. The short channels were not included in subsequent statistical analyses.

Twenty channels were recorded at three wavelengths (780 nm, 805 nm and 830 nm), and the optical densities obtained from the FOIRE-3000 resulted in 60 time series. The log_10_-based optical density data were converted to natural logarithm-based absorbances, and each channel was referenced to the mean of each complete time series (~12 min duration). The absorbance data were then low-pass filtered to approximately 0.66 Hz using a moving average with a window length corresponding to 1.5 s. All 60 time series were smoothed separately. The concentration changes of O_2_Hb and HHb were calculated with the modified Beer–Lambert law at the following wavelengths: λ_1_ = 780 nm, λ_2_ = 805 nm and λ_3_ = 830 nm [48]. Time series of the concentration changes in O_2_Hb and HHb were obtained for each channel separately. For the calculation, we used the molar absorption coefficients of Prahl [49] and the differential path length factor that was calculated from a generalizing equation [50].

To remove movement artifacts, the movement artifact removal algorithm (MARA) was employed [51]. The parameters for MARA, including the moving average window length *L*, threshold *T* and reconstruction parameter *p*, were individually selected for each channel and O_2_Hb and HHb signal. We used a slightly different version of MARA, which did not use spline interpolation, but rather smoothed a running 2nd order least squares polynomial model (with *p* being the moving average window length) for the artifacts and subtracting this fit to preserve the high frequency information [52]. The parameter *p* was always set to seven samples, which corresponded to the preservation of the artifact data above approximately 1 Hz. The movement artifact removal was done in the following three steps: (1) trained raters analyzed the data; (2) experienced MARA users verified the results of the first step; and (3) a senior fNIRS data processing expert verified the cases marked as ambiguous. After the data were passed to MARA, they were again low-pass filtered (cut-off frequency, 0.2 Hz) with a 2nd order Chebyshev infinite-impulse-response filter.

To calculate the block response, a general linear model (GLM) was applied. We used a set of 129 Gaussian base functions with a standard deviation of 0.5 s that were separated by 0.5 s. The 129 base functions covered a time range of 65 s. The tasks were analyzed within a time window ranging from 5–45 s. The preceding 5 s were defined as the baseline, which was subtracted from the signal. The GLM followed the description in Gagnon et al. [47]. We excluded blocks from the GLM when the percentage of correct responses was less than 80% and 50% for the control and experimental conditions, respectively. Participants with less than three remaining blocks within each condition were excluded from further analyses. The remaining blocks were averaged by the GLM model, and a block average (subject-specific hemodynamic response function) was obtained for each variable (O_2_Hb and HHb), subject and task. To reduce the amount of data for statistical analysis, the sampling frequency was reduced to 0.2 Hz after averaging 5 s-long segments. For the statistical analysis, the mean concentration changes in O_2_Hb and HHb relative to baseline were calculated separately for each participant in both tasks (WM and DD), both conditions (control and experimental) and each channel (Channels 1–16). All statistical analyses were conducted with R [53]. To adjust for multiple testing, the Bonferroni–Holm correction [54] was applied for post hoc pairwise comparisons.

### 2.4. Time Course of the Study

In the first test session, over 250 participants were screened for mental ability. The participants who qualified for either the LA or HA mental ability group performed the DD task and visuospatial WM task in the second session. The order of the tasks was counterbalanced across the participants. The fNIRS measurements were performed in the third session. The average interval between the sessions was two weeks. 

## 3. Results

### 3.1. Behavioral Data

Table 1 shows the descriptive statistics of the performance measures obtained in the visuospatial WM and DD tasks for the total sample, as well as the LA and HA groups. In the WM task, the participants reached a mean ± standard deviation percentage of correct responses of 99.0 ± 2.4% in the control condition and 88.7 ± 7.2% in the experimental condition (*t*(42) = −9.42, *p* < 0.001, *d* = −1.95). In the DD task, the mean ± standard deviation percentage of correct responses was 94.6 ± 2.9% in the control condition and 78.0 ± 9.8% in the experimental condition (*t*(42) = −10.08, *p* < 0.001, *d* = −2.30). These findings clearly indicated that the task demands were significantly increased in the experimental condition compared with the control condition in both tasks. Furthermore, the percentages of correct responses did not differ significantly between the LA and HA groups in either task (see Table 2). Thus, we accomplished our goal of obtaining equivalent levels of subjective task difficulty for the participants in the two mental ability groups. 

### 3.2. fNIRS Data

Definition of the region of interest (ROI): We applied an exploratory and data-driven approach to investigate possible ROIs [55]. We first selected channels with which we could observe the typical patterns of hemodynamic responses during functional activation (increase in O_2_Hb and decrease in HHb) [41]. For the frontal cortex, this pattern enhances the probability that the signal does not contain systemic changes [41,56] and indicates good signal quality [57]. We therefore selected only the channels in which O_2_Hb and HHb were negatively correlated in both conditions (control and experimental) and both tasks (WM and DD). We applied this criterion to both tasks to ensure that any effects would be detected with the highest probability. This criterion was met by Channels 1, 2 and 3. Plots of the time course of O_2_Hb and HHb for all three channels, both conditions (active control and experimental) and both tasks (WM and DD) can be found in the Supplementary Materials (Figure S2). All three channels were located next to each other in a region covering Brodmann Area (BA) 8, and thus, they defined our ROI. The Montreal Neurological Institute coordinates (x, y, and z) of Channels 1–3 were (−8, 49, 47), (−26, 43, 47) and (−40, 25, 47), respectively. The mean concentration changes in O_2_Hb and HHb relative to baseline of Channels 1–3 were averaged and provided the value for our ROI. Within this ROI, the negative correlations between O_2_Hb and HHb were significant for both tasks and both conditions (*r*_WM control_ = −0.56, *r*_WM experimental_ = −0.75, *r*_DD control_ = −0.74, *r*_DD experimental_ = −0.78 (all *p*s < 0.001, two-tailed)). A projection of the ROI on a standard brain is depicted in Figure 2.

**Figure 3:**
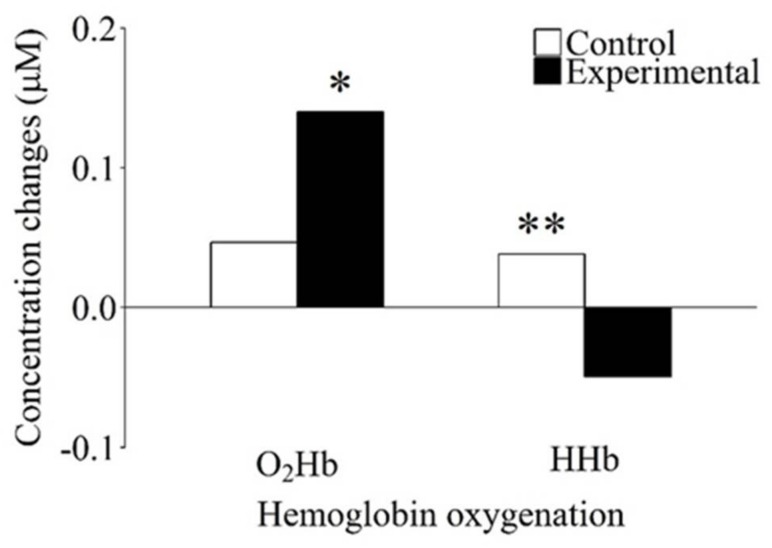
Differences in concentration changes for oxygenated (O_2_Hb) and deoxygenated (HHb) hemoglobin as a function of condition for the working memory task. HHb concentration was significantly lower in the experimental than in the control condition. Within the experimental condition, there was a significant difference in concentration change between O_2_Hb and HHb. * significantly different from HHb concentration change in the experimental condition (*p* < 0.05); ** significantly different from HHb concentration change in the experimental condition (*p* < 0.01).

To examine whether the changes in functional activation observed during the performance of the WM task were specific for WM-related processes and not related to more general subsidiary processes, we investigated the differences in the Hb oxygenation concentration changes observed in the control and experimental conditions in the WM task by conducting a two-way analysis of variance with the within-subject factors of Hb oxygenation (O_2_Hb and HHb) and condition (control and experimental). This analysis yielded no significant main effects (Hb oxygenation, *F*(1, 42) = 2.84, *p* = 0.099, *η_p_*^2^ = 0.063; condition, *F*(1, 42) = 0.01, *p* = 0.920, *η_p_*^2^ < 0.001). Instead, a significant interaction of Hb oxygenation and condition was found (*F*(1, 42) = 4.41, *p* = 0.042, *η_p_*^2^ = 0.095). Post hoc pairwise comparisons revealed a significant decrease in the relative concentration of HHb in the experimental condition compared to the control condition (*t*(42) = 3.82, *p* = 0.002, *d* = 0.59). In addition, the O_2_Hb relative concentration was significantly increased compared to the HHb relative concentration (*t*(42) = 2.61, *p* = 0.037, *d* = 0.70) in the experimental condition (see Figure 3 and Table 3).

**Figure 4:**
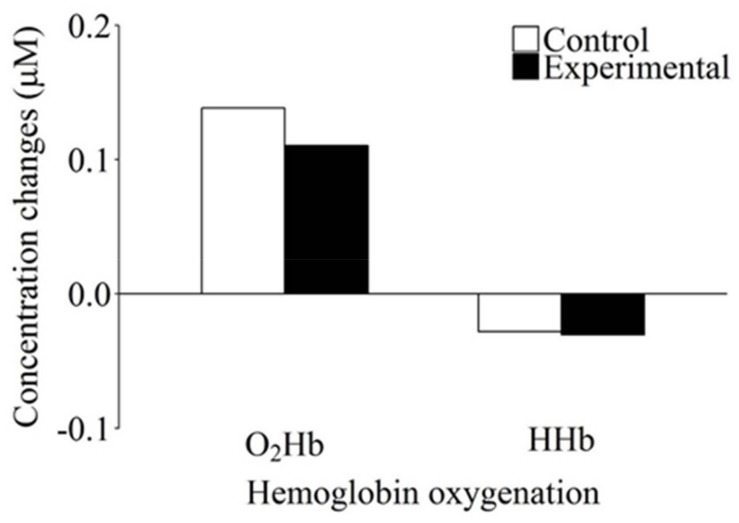
Concentration changes for oxygenated (O_2_Hb) and deoxygenated (HHb) hemoglobin as a function of condition for the duration discrimination task. Neither O_2_Hb nor HHb showed a significant difference in hemoglobin oxygenation concentration change between the control and the experimental condition.

To confirm whether the increased functional activation observed in the experimental condition represented WM task-specific processes and not general effects of increased task demands, we evaluated whether the differences in the O_2_Hb and HHb concentration changes that occurred as a function of fNIRS condition would also be observed in the DD task. For this purpose, a two-way analysis of variance that was identical to the one applied for the visuospatial WM task was conducted. This analysis did not yield any indication of concentration changes in Hb oxygenation. Neither the main effects of Hb oxygenation (*F*(1, 42) = 3.14, *p* = 0.084, *η_p_*^2^ = 0.070) or condition (*F*(1, 42) = 0.57, *p* = 0.453, *η_p_*^2^ = 0.013), nor the interaction of these factors (*F*(1, 42) = 0.11, *p* = 0.743, *η_p_*^2^ = 0.003) was statistically significant (see Figure 4 and Table 3).

As a final step, we examined if mental ability level effectively moderated the concentration changes in Hb oxygenation observed in the WM task. For this purpose, a three-way analysis of variance with Hb oxygenation (O_2_Hb and HHb) and condition (experimental and control) as the two within-subject factors and mental ability (LA and HA) as the between-subjects factor was performed. This analysis yielded no significant main effects of Hb oxygenation (*F*(1, 41) = 2.75, *p* = 0.105, *η_p_*^2^ = 0.063), condition (*F*(1, 41) = 0.21, *p* = 0.884, *η_p_*^2^ = 0.001) or mental ability (*F*(1, 41) = 0.49, *p* = 0.486, *η_p_*^2^ = 0.012). However, a significant interaction of Hb oxygenation and condition (*F*(1, 41) = 5.33, *p* = 0.026, *η_p_*^2^ = 0.115), as well as a statistically significant three-way interaction of all combined factors (*F*(1, 41) = 7.14, *p* = 0.011, *η_p_*^2^ = 0.148) was observed. No significant two-way interactions were found for condition and mental ability (*F*(1, 41) = 3.32, *p* = 0.076, *η_p_*^2^ = 0.075) or Hb oxygenation and mental ability (*F*(1, 41) = 0.48, *p* = 0.492, *η_p_*^2^ = 0.012).

**Figure 5:**
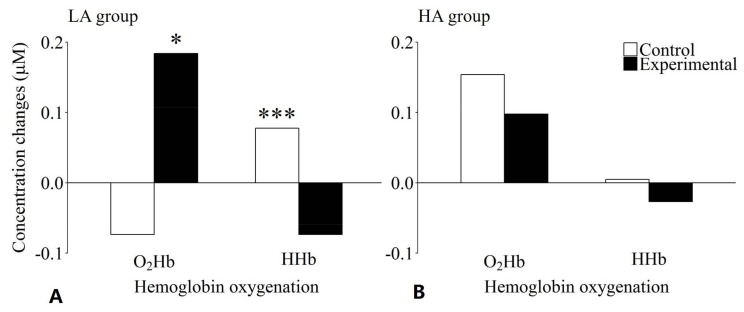
Effects of level of mental ability and condition on hemoglobin oxygenation concentration changes for the working memory task in the lower (**A**) and the higher (**B**) mental ability group. The lower mental ability group showed significantly increased O_2_Hb and significantly decreased HHb concentrations in the experimental compared to the control condition. No significant differences could be observed for the higher mental ability group. * Significantly different from O_2_Hb concentration change in the control condition (*p* < 0.05); *** significantly different from HHb concentration change in the experimental condition (*p* < 0.001).

To better assess the significant three-way interaction, two separate two-way analyses of variance were conducted with the factors of Hb oxygenation and condition for the LA and HA groups. For the LA group, no significant main effects of Hb oxygenation (*F*(1, 20) = 0.36, *p* = 0.555, *η_p_*^2^ = 0.018) or condition (*F*(1, 20) = 2.62, *p* = 0.121, *η_p_*^2^ = 0.116) were revealed, whereas the interaction between these two factors was significant (*F*(1, 20) = 16.67, *p* = 0.001, *η_p_*^2^ = 0.455). The post-hoc pairwise comparisons indicated that the relative concentration of O_2_Hb was significantly higher in the experimental condition than in the control condition (*t*(20) = 3.30, *p* = 0.039, *d* = 0.68). A difference was observed in the opposite direction for HHb: the relative HHb concentration was significantly lower in the experimental condition than in the control condition (*t*(20) = 4.93, *p* < 0.001, *d* = 0.97 (see Figure 5A)). In contrast to the LA group, neither a statistically significant main effect of Hb oxygenation (*F*(1, 21) = 3.79, *p* = 0.065, *η_p_*^2^ = 0.153) or condition (*F*(1, 21) = 1.14, *p* = 0.298, *η_p_*^2^ = 0.51), nor a statistically significant interaction between these two factors (*F*(1, 21) = 0.54, *p* = 0.819, *η_p_*^2^ = 0.003) was found for the HA group (see Figure 5B).

## 4. Discussion

The present study investigated whether fNIRS was useful for measuring WM-specific process-induced functional activation in the prefrontal cortex that was independent of competing processes that were less specific to WM functioning. In particular, we examined whether the differences in Hb oxygenation concentration changes observed during the performance of the WM task were due to specific WM capacity-related processes rather than to subsidiary processes, such as motor processes or perceptual encoding (e.g., [58,59]), or more general processes, such as those related to increased task demand. To achieve this goal, we compared the Hb oxygenation concentration changes that were observed during task performance in the experimental condition with those observed during the active control condition in both a WM and a DD task.

Initially, an ROI was identified. Only three out of the 16 channels met our strict criterion of a negative correlation between O_2_Hb and HHb in the experimental and control conditions of both tasks. These three channels defined our ROI above BA 8 in the left hemisphere. While Channels 1 and 2 covered the left superior frontal gyrus, Channel 3 was located above the left middle frontal gyrus. Despite the limited size of our holder, the data-driven approach for defining our ROI resulted in an area that has been shown to be relevant for visuospatial WM functioning in previous studies. For example, Boisgueheneuc et al. [60] have reported that patients with a lesion in BA 8 exhibit WM deficits, especially in visuospatial tasks, compared to patients with lesions that did not involve the superior frontal gyrus and healthy controls. However, not only different WM task modalities (e.g., visuospatial and verbal), but also distinct WM processes (e.g., encoding, storing and retrieval of information) are represented by different functional activation patterns in the brain. For example, these functional activation patterns differ depending on whether the localization or identity of a stimulus needs to be memorized or whether the stimulus must be reproduced on retrieval or simply verified. Rottschy et al. [11] have shown that memory for stimulus location and the reproduction of a memorized stimulus lead to increased functional activation in the posterior superior frontal gyrus. This finding is consistent with the outcomes of the present study in which the WM task also required the storing and subsequent reproduction of the localization of black squares that was accompanied by functional activation in BA 8.

In order to more clearly attribute the functional activation observed during the processing of the WM task to task-specific processes, we compared an active control condition that required only general subsidiary processes with an experimental condition that additionally required WM-specific processes. In the experimental condition, O_2_Hb increased and HHb decreased, which corresponds to the typical pattern of hemodynamic responses observed during functional activation [41,61]. Comparison of the conditions clearly indicated additional resource consumption by the WM-specific task demands in the experimental condition.

This finding was in line with the outcomes of previous fNIRS studies that applied multiple levels of task difficulty. For example, Causse and colleagues [62] investigated changes in O_2_Hb and HHb in the prefrontal cortex as a function of task difficulty in the spatial WM task in the Cambridge Neuropsychological Test Automated Battery. Their results revealed that fNIRS was sensitive to variations in task difficulty as O_2_Hb increased and HHb decreased with the increasing number of localizations that needed to be memorized. Similar patterns of O_2_Hb and HHb changes have been reported by several other studies applying verbal n-back tasks with letters as stimuli. The task demands were experimentally varied by using either 1-, 2- or 3-back [8,9,63] or 0-, 1-, 2- or 3-back conditions [64,65]. These studies also confirmed that increasing task difficulty resulted in increased O_2_Hb and decreased HHb concentrations.

Despite these consistent findings for task difficulty, it remains unclear whether this typical functional activation pattern is caused by WM-specific processes or is due to the higher degree of task demand caused by the more difficult conditions and is independent of the specific task under investigation. In order to confirm whether the increased functional activation observed in the experimental condition in the present study was caused by WM task-specific processes and did not reflect a general effect of a task-independent increase in task demand, we implemented DD as a supplementary task that was functionally independent of WM capacity. DD of extremely brief intervals in the range of milliseconds is processed subcortically [25,29] and should, therefore, not cause any changes in prefrontal O_2_Hb and HHb. If the differences in Hb oxygenation between the control and experimental conditions that were detected with the WM task were predominantly task-unspecific, thus indicating that the changes caused by the experimentally-induced increase in general task demand would be produced by any given task, a similar pattern should be observed in the differences in Hb oxygenation in both the WM and DD tasks. If, however, the Hb oxygenation differences were only observed during the processing of the WM task and not during the performance of the DD task, the measured differences in the concentration changes might be directly related to WM capacity. The complete absence of any changes in O_2_Hb and HHb in the experimental and control conditions in the DD task clearly indicated that the pattern of changes observed with the WM task reflected a hemodynamic response that was highly specific to the experimentally-increased visuospatial WM capacity-related task demands. Thus, to the best of our knowledge, the present study is the first to provide direct evidence of the sensitivity of fNIRS for identifying hemodynamic responses specific for visuospatial WM capacity by utilizing a WM-unrelated control task.

Comparisons between different types of tasks (e.g., WM and DD) and different task conditions (e.g., active control and experimental) are only conclusive if prerequisites were created that enabled their comparison. We accomplished this by developing a control and experimental condition for each task and applying an adaptive experimental approach in both tasks. The goal was to keep the items in the control condition in both tasks as simple as possible so that no specific task demands were required. At the behavioral level, this was evident because virtually all items were correctly solved by all participants. In contrast, the items in the experimental conditions in the WM and DD tasks should contain additional specific task demands. The specific task demands of the WM and DD tasks rendered the experimental condition in both tasks more difficult than the respective control conditions. In both tasks, the percentage of correct answers in the control condition was significantly higher than that in the experimental condition.

With the adaptive approach, we established that all individuals were presented with task demands that were subjectively equally difficult in both tasks. At the behavioral level, this was demonstrated because the two mental ability groups did not differ in the percentages of correct responses in both types of tasks and both task conditions. With 78% correct responses, the DD task was somewhat more difficult than the WM task, which had 89% correct responses. This should have facilitated the occurrence of a general effect of a task-independent increase in task demand in the DD compared to the WM task. Based on these considerations, the complete lack of any changes in Hb oxygenation in the experimental condition in the DD task could not be attributed to the task demand level being too low to elicit a hemodynamic response relative to the WM task.

In the final step, the potential moderating effects of individual differences in mental ability on the observed WM-specific changes of Hb oxygenation were investigated. For this purpose, we compared the WM-specific changes in Hb oxygenation in the LA and HA groups. It should be noted that our LA group consisted of individuals with an average mental ability (IQ ranging from 90–112), while our HA group comprised individuals who were considerably above average mental ability (IQ ranging from 130–145). The analysis revealed a clear-cut moderating effect of mental ability level. In the LA group, the WM-specific task demands of the experimental condition induced the typical functional activation pattern, which was indicated by an O_2_Hb increase and HHb decrease (e.g., [41,61,66]). This pattern of hemodynamic changes was indicative of functional activation in the experimental condition compared to the control condition. In contrast, the HA group showed an almost identical pattern of Hb concentration changes in both the control and experimental conditions without any indication of functional activation.

These Hb oxygenation patterns that differed as a function of individual level of mental ability were in line with the results of various neuroimaging studies. Early PET and single-photon emission computed tomography studies investigating the link between mental ability and functional activation have reported less functional activation in individuals with higher mental abilities during the processing of cognitive tasks compared with individuals with lower mental abilities (e.g., [32,67,68]). Subsequent fMRI studies further differentiated these findings by systematically varying task demand [69,70,71,72,73]. These studies revealed that the described effects occurred, especially with tasks with low to medium difficulties. More recently, this reciprocal action between cognitive task demand and level of mental ability on functional activation was confirmed by an fNIRS study [74]. When cognitive decision-making tasks were performed, low-conflict decisions (i.e., lower cognitive task demands) produced a stronger hemodynamic response in the frontal cortex in participants with low mental abilities compared with participants with high mental abilities. This pattern of functional activation, however, did not hold for high-conflict decisions with enhanced cognitive task demands.

Overall, the findings of these previous studies revealed that individuals with lower mental abilities showed stronger functional activation compared to individuals with higher mental abilities during the processing of cognitive tasks with low to moderate levels of task difficulty. At the same time, the performances on these cognitive tasks confirmed the differences between the groups: the individuals with lower mental abilities consistently performed more poorly than the individuals with higher mental abilities did during the processing of the same WM task. This latter finding clearly indicated that a given cognitive task with low difficulty was subjectively more demanding for individuals with lower mental abilities compared to individuals with higher mental abilities. Thus, it is reasonable to assume that this inequality in subjective task demand may have caused the observed differences in functional activation between the low and high mental ability groups.

Taking this background into account, it appeared reasonable to assume that the observed differences in functional activation would disappear when the participants were required to process a cognitive task with the same level of subjective task demand. In order to ensure that all individuals experienced an equivalent level of subjective task demand, we applied an adaptive approach in the present study. With this experimental approach, we ensured that each participant was presented with the same visuospatial WM task with task difficulty individually adjusted to obtain a virtually-identical level of subjective task demand irrespective of his/her individual level of mental ability.

As a result, both the LA and HA groups achieved correct responses on the order of 88%, which was indicative of a cognitive task of low difficulty. This low level of task difficulty corresponded to the difficulty level of the WM tasks used in the previous studies, in which a differential effect in functional activation was demonstrated between the mental ability groups. Rather unexpectedly, however, we found that the difference in functional activation still existed despite the virtually identical subjective task demands for both mental ability groups. Therefore, it must be ruled out that the observed differences in functional activation between the LA and HA groups were caused by differences in subjective task demands, as has been suggested by the results of previous studies. Rather, individuals with lower mental abilities seem to utilize more cortical oxygen compared with high ability individuals while solving an easy visuospatial WM task that was subjectively adjusted for cognitive task demand across subjects. This highly surprising finding may indicate that qualitatively different cognitive processes were adopted by the LA and HA individuals to solve the task.

In order to provide converging evidence of the validity of this finding, additional samples of LA and HA participants should be tested in future studies. Furthermore, additional studies are required to examine whether a similar moderating effect of mental ability on functional activation can also be demonstrated for other WM tasks. 

## 5. Conclusions

Taken together, our findings clearly indicated that that hemodynamic responses recorded by fNIRS were sensitive to visuospatial WM capacity-related processes. Furthermore, the functional activation observed proved to be specific to the WM processes under investigation rather than to general effects of increased task demand. Finally, we provided converging evidence for a moderating effect of the individual level of mental ability on the hemodynamic responses. Because this effect could not be attributed to differences in subjective task demand, our findings suggest that qualitatively different processes were involved in visuospatial WM processing in LA and HA individuals.

## Figures and Tables

**Table 1:** Mean (M) and standard deviation (SD) of visual-spatial working memory span (WM) and duration discrimination threshold (DD) for the total sample (total, *n* = 43) as well as for the lower (LA, *n* = 21) and higher (HA, *n* = 22) mental ability groups.

Task	Total		LA		HA	
	M	SD	M	SD	M	SD
WM (span)	5.9	0.96	5.4	0.75	6.4	0.91
DD (threshold in ms)	28.7	10.61	30.2	11.15	27.3	10.11

**Table 2:** Mean (M) and standard deviation (SD) of percentages of correct responses in the working memory (WM) and duration discrimination (DD) tasks and both fNIRS conditions (experimental and control) for the total sample (total, *n* = 43) as well as for the lower (LA, *n* = 21) and higher mental ability (HA, *n* = 22) groups separately. *t*-tests and effect size estimates (Cohen’s *d*) comparing the two groups.

Task	Condition	Total		LA		HA				
		M	SD	M	SD	M	SD	*t*(41)	*p*	*d*
WM	control	99.0	2.38	99.1	2.23	98.9	2.56	0.25	0.804	0.08
	experimental	88.7	7.15	89.0	7.22	88.4	7.23	0.27	0.791	0.08
DD	control	94.6	2.85	94.5	2.86	94.7	2.91	−0.28	0.783	−0.09
	experimental	78.0	9.84	79.0	9.98	77.1	9.85	0.63	0.530	0.19

**Table 3:** Mean (M) and standard deviation (SD) of oxygenated (O_2_Hb) and deoxygenated (HHb) hemoglobin concentration changes as a function of fNIRS condition (control and experimental) in the working memory (WM) and duration discrimination (DD) tasks for the total sample (Total, *n* = 43), as well as for the lower (LA, *n* = 21) and higher (HA, *n* = 22) mental ability groups.

Task	Hemoglobin Oxygenation	Condition	Total		LA		HA	
			M	SD	M	SD	M	SD
WM	O_2_Hb	control	0.05	0.38	−0.07	0.35	0.15	0.39
		experimental	0.14	0.35	0.18	0.39	0.09	0.31
	HHb	control	0.04	0.14	0.08	0.16	0.00	0.12
		experimental	−0.05	0.15	−0.07	0.15	−0.03	0.16
DD	O_2_Hb	control	0.14	0.48	0.10	0.35	0.17	0.59
		experimental	0.11	0.47	0.07	0.35	0.14	0.57
	HHb	control	−0.03	0.16	−0.01	0.14	−0.05	0.18
		experimental	−0.03	0.19	−0.02	0.10	−0.04	0.25

## Supplementary Materials

The following are available online at http://www.mdpi.com/2076-3425/8/4/62/s1: Figure S1: Image of probe holder mounted on the left forehead, Figure S2: Grand averages and confidence intervals of evoked hemodynamic concentration changes across all subjects for Channels (CH) 1–3. Red line = oxygenated hemoglobin, blue line = deoxygenated hemoglobin, each with the 95% confidence interval.
