# Supplementary material for: The Validity of Functional Near-Infrared Spectroscopy Recordings of Visuospatial Working Memory Processes in Humans
Source: Brain Sci. 2018 Apr 5;8(4):62. doi: 10.3390/brainsci8040062 (PMC5924398; doi:10.3390/brainsci8040062)
Supplement: Supplementary file 1 [file brainsci-08-00062-s001.pdf]

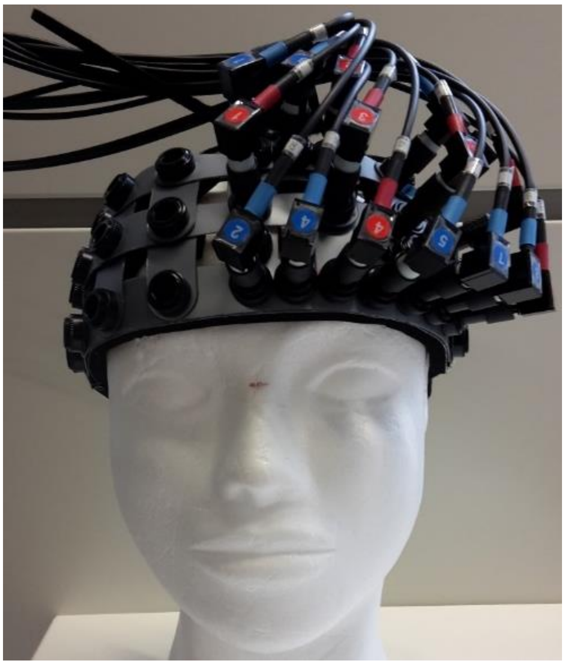

Figure S1. Image of probe holder mounted on the left forehead.

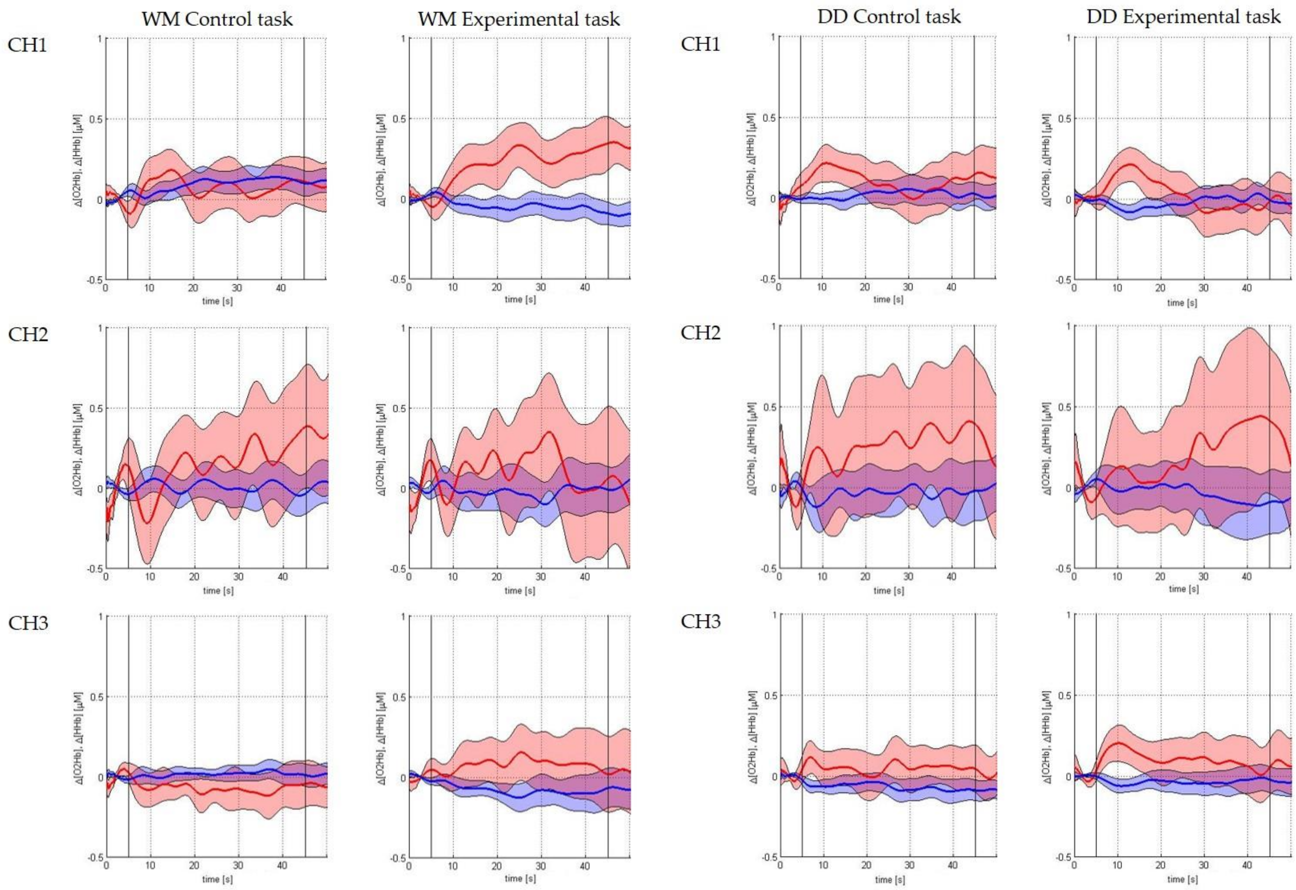

Figure S2. Grand averages and confidence intervals of evoked hemodynamic concentration changes across all subjects for Channels (CH) 1 to 3. Red line = oxygenated hemoglobin, blue line = deoxygenated hemoglobin, each with 95% confidence interval.
